# Construction of a Biosensor Based on a Combination of Cytochrome *c*, Graphene, and Gold Nanoparticles

**DOI:** 10.3390/s19010040

**Published:** 2018-12-22

**Authors:** Chenxing Guo, Jianfang Wang, Xianzhe Chen, Yujiao Li, Lifang Wu, Jin Zhang, Cheng-an Tao

**Affiliations:** 1 Key Laboratory of Environmentally Friendly Chemistry and Application of Ministry of Education, College of Chemistry, Xiangtan University, Xiangtan 411105, China; gcx@carestech.cn (C.G.); wulifangyou@163.com (L.W.); 2 College of Liberal Arts and Science, National University of Defense Technology, Changsha 410073, China; chenxianzhe13@nudt.edu.cn (X.C.); liyujiao@nudt.edu.cn (Y.L.)

**Keywords:** biosensor, cytochrome *c*, graphene, gold nanoparticles, hydrogen peroxide, direct electrochemistry

## Abstract

A biosensor based on a combination of cytochrome *c* (Cyt *c*), electrochemical reduced graphene oxides (ERGO), and gold nanoparticles (AuNPs) on a glassy carbon electrode (GCE) was fabricated. The proposed biosensor electrode was denoted as GCE/ERGO-Nafion/AuNPs/Cyt *c*/Nafion, where ERGO-Nafion was deposited by dropping graphene oxides-Nafion mixed droplet first and following electrochemical reduction, AuNPs were directly deposited on the surface of the ERGO-Nafion modified electrode by electrochemical reduction, and other components were deposited by the dropping-dry method. The effect of the deposition amount of AuNPs on direct electrochemistry of Cyt *c* in the proposed electrode was investigated. The hydrogen peroxide was taken to evaluate the performance of the proposed biosensor. The results showed that the biosensor has great analytical performance, including a high sensitivity, a wide linear range, a low detection limit, and good stability, reproducibility, and reliability.

## 1. Introduction

Cytochrome *c* (Cyt *c*) is a water soluble heme-containing protease that is an important redox protein found between the inner and outer membranes of mitochondria in various animals [1,2,3,4]. It is an important electron carrier, which has two forms, a reduced state containing divalent ferrous iron, and an oxidation state containing ferric iron [5,6]. Cytochrome *c* can be transformed into each other in these two states, and is a biomacromolecule with both high catalytic and sensory properties, so it can be widely used in sensor research [7,8]. The heme of cytochrome *c* is its potential active center, but the heme is surrounded by a protein shell, which hinders contact with the outside world. Moreover, Cyt *c* can be directly and strongly adsorbed to the surface of the varied electrodes, such as gold, silver, diamond, Pt, and carbon nanotubes [9,10,11]. However, Cyt *c* was found to be denatured quickly during voltammetric measurements and the electron transfer kinetics of Cyt *c* are found to be sluggish at the above-mentioned electrodes, suggesting that protein variability occurs and the activity is lost. It is important for the application of Cyt *c* in electronchemical fields to search for materials that can be used to promote electron transfer [7,12,13,14].

Graphene is a new type of two-dimensional carbon sheet with a single layer of *sp*^2^ carbon atoms [15,16,17]. It has been demonstrated that graphene has rapid electron transmission, excellent conductivity, high specific surface area, and high electrocatalytic activity [17,18]. However, graphene is hydrophobic and easy to agglomerate, even graphitization due to Van der Waals forces and π-π stacking, which greatly limits its application in biosensors [16]. Graphene oxide (GO), a derivative of graphene, possesses various oxygen-containing functional groups, such as hydroxyl, epoxy, carboxylic acid groups, etc. [19,20], which induced the good water-solubility and biocompatibility of GO [21]. Therefore, GO is more favorable to be used as the precursor of graphene, and the reducing procedure can avoid the agglomeration problem. More importantly, there are still many boundary points, remaining functional groups, and structural defects remaining in reduced GOs, which not only can promote the total electron transfer in the reaction process, thereby achieving the direct electrochemical behavior of the enzyme sensor, but also increase the biocompatibility of graphene, and prevent biomacromolecules losing their biological activity after loading biomacromolecules onto the graphene surface [22,23]. In this regard, Cyt *c* on various graphene based electrodes has been investigated [14,22,23,24,25].

Gold nanoparticles (AuNPs) have also widely served as electrode materials for the development of biosensors [26,27,28,29] due to their novel properties, such as a superior specific area, fine conductivity, perfect catalytic activity, and nice biocompatibility. Moreover, it was found that the electrocatalytic synergy of graphene and AuNPs can extremely improve the performance of the resultant electrochemical sensor [18,25,30,31]. Song et al [25] explored the synergic electrocatalytic effect of graphene and AuNPs on the direct electrochemistry of Cyt *c*. They developed a biosensor electrode by entrapping Cyt *c* in thin films of room temperature ionic liquid (RTIL) containing nanocomposites of poly (diallyldimethylammonium chloride) (PDDA)-graphene nanosheets-gold nanoparticles (PDDA-Gp-AuNPs) at a 11-mercaptoundecanoic acid-6-mercapto-1-hexanol modified gold electrode (MUA-MCH/Au). They synthesized the PDDA functionalized graphene nanosheets and AuNPs in advance, and then deposited them on the surface of the MUA-MCH/Au electrode to form a Cyt *c*/RTIL-PDDA-Gp-AuNPs/MUA-MCH/Au electrode. However, the process was complicated and time-consuming.

**Figure 1:**
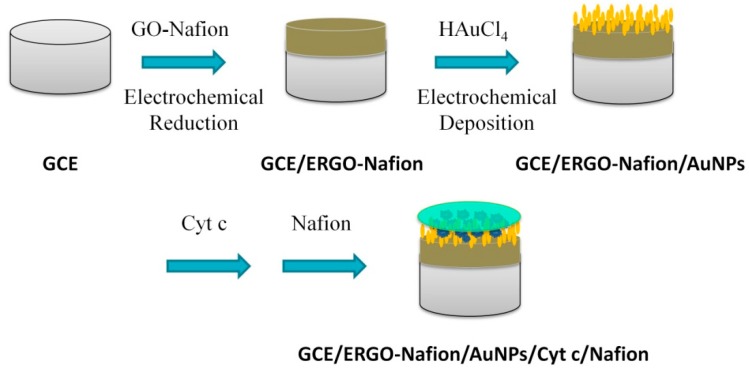
Step-by-step construction of a GCE/ERGO-Nafion/AuNPs/Cyt c/Nafion electrode.

In this study, we proposed an improved procedure to fabricate a novel biosensor based on a combination of Cyt *c*, electrochemical reduced graphene oxides (ERGO), and AuNPs on a glass carbon electrode (GCE). The GCE/ERGO-Nafion/AuNPs/Cyt *c*/Nation electrode (‘/’ means which were used to modify electrodes in successive steps, ‘-’ represents which are mixed first, and then used to modify electrodes) were deposited stepwisely by simple dropping-dry method (Figure 1). Both ERGO-Nafion and AuNPs were rapidly prepared on the surface of the electrode by electrochemical reduction. Additionally, the effect of the deposition amount of gold nanoparticles on the direct electrochemistry of Cyt *c* in the proposed electrode was explored. The rapid and accurate analysis of hydrogen peroxide (H_2_O_2_) was of great significance in biology, clinical control, the food industry, and environmental protection. The proposed electrode could be utilized for electrocatalytic reduction as well as amperometric determination of H_2_O_2_. The detection limit and linear range for H_2_O_2_ were 1.1 μM and 0.01 mM to 3.5 mM, respectively. Moreover, the biosensor exhibited high sensitivity and stability with good reproducibility and reliability.

## 2. Materials and Methods

### 2.1. Material and Apparatus

The GO was prepared and characterized as described in our previous works [32,33]. Nafion (5% ethanol solution) was purchased from J&K Chemical (Beijing, China). Chloroauric Acid (HAuCl_4_, AR), sulfuric acid (H_2_SO_4_, AR), H_2_O_2_ (30%), glucose (AR), methanol (AR), ethanol (AR) and potassium chloride (KCl), potassium ferricyanide (K_3_[Fe(CN)_6_], AR), potassium ferrocyanide (K_4_[Fe(CN)_6_], AR), sodium dihydrogen phosphate (NaH_2_PO_4_, AR), and disodium hydrogen phosphate (Na_2_HPO_4_, AR) were purchased from Sinopharm Chemical (Beijing, China). Phosphate buffer saline (0.2 M PBS, pH = 7.0) was prepared by mixing 0.2 M NaH_2_PO_4_ and 0.2 M Na_2_HPO_4_. Cytochrome *c* from bovine heart (Cyt *c*, ≥ 95%) was obtained from aladdin chemical (Shanghai, China). All chemicals were used as received without further purification. All solutions were prepared using ultrapure water. 

Fourier transform infrared (FT-IR) spectra were obtained using a Spectra Two (PerkinElmer) spectrophotometer within the spectral range of 4000 cm^−1^ to 500 cm^−1^ with an attenuated total reflection (ATR) accessory. Scanning electron microscopy (SEM) images were obtained using a Hitachi S-4800 electron microscope. The samples were sputtered with a thin layer of Au prior to imaging except that which contained gold nanoparticles.

All electrochemical experiments were carried out on a CHI660A electrochemical analyzer (Chen Hua Instruments, Shanghai, China) using a three-electrode system, where a bare or modified glass carbon electrode (Ф = 3 mm) served as the working electrode, an Ag/AgCl (3M KCl solution) electrode was adopted as the reference electrode, and a platinum wire was used as the auxiliary electrode, respectively. The cyclic voltammetric (CV) experiments were performed in a quiescent solution. Electrochemical impedance spectroscopy (EIS) was run in a 0.01 M KCl solution containing 10 mM of K_3_[Fe(CN)_6_]/K_4_[Fe(CN)_6_] (1:1) at frequencies ranging from 0.01 Hz to 100 kHz at open circle potential. The amperometric experiments were performed in a continuously stirred solution using a magnetic stirrer (300 rpm).

### 2.2. Preparation of GCE/ERGO-Nafion/AuNPs/Cyt c/Nafion Electrode

The GCE electrode was polished with 1.0 μM, 0.3 μm, and 50 nm γ-Al_2_O_3_ successively, then rinsed with deionized water, and sonicated in deionized water and ethanol for 5 min. Finally, it was dried by N_2_ and placed at 4 °C for use. The GO (10 mg) was added to 10 mL of ethanol containing 0.2% Nafion under ultrasonic for 30 min to prepare a mixture of GO-Nafion. Then, 6 μL of GO-Nafion mixed droplet was placed on the surface of the GCE. The obtained electrode was dried at room temperature, then reduced by the CV method in the range of −0.8V to −1.7V in 0.5 M KCl solution at a scan rate of 50 mV/s to achieve the GCE/ERGO-Nafion electrode. Then, gold nanoparticles (AuNPs) were electrodeposited by CV in the range of −0.55 to −0.05 V at a scan rate of 10 mV/s in a solution of 0.5 M H_2_SO_4_ containing 0.4 mg/mL of chloroauric acid, and a GCE/ERGO-Nafion/AuNPs electrode was fabricated. Next, 7.5 μL of 1 mg/mL cytochrome c was added dropwise to the surface of GCE/ERGO-Nafion/AuNPs, and finally 6 μL of 0.2% Nafion solution was added dropwise to obtain a GCE/ERGO-Nafion/AuNPs/Cyt *c*/Nafion electrode. 

For comparison, GCE/Cyt *c*/Nafion, GCE/AuNPs/Cyt *c*/Nafion, and GCE/ERGO-Nafion/Cyt *c*/Nafion electrodes were prepared using a similar procedure.

## 3. Results

### 3.1. Construction of GCE/ERGO-Nafion/AuNPs/Cyt c/Nafion Electrode

**Figure 2:**
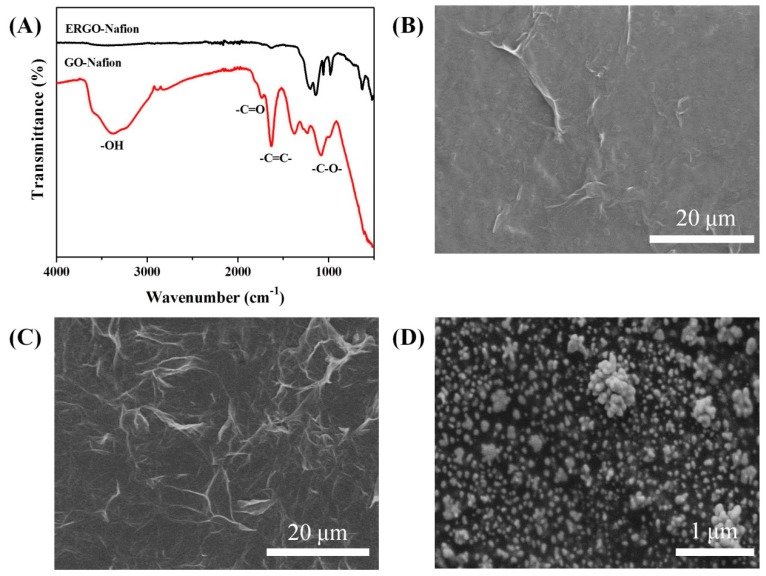
(**A**) FTIR spectra of GO-Nafion and ERGO-Nafion; SEM images of (**B**) GO-Nafion, (**C**) ERGO-Nafion, and (**D**) AuNPs on ERGO-Nafion.

The GCE/ERGO-Nafion/AuNPs/Cyt *c*/Nafion electrodes were sequentially constructed on the GCE electrodes in the order of ERGO-Nafion, AuNPs, Cyt *c*, and Nafion. First, the GO-Nafion mixed solution was dropped onto the surface of the GCE electrode, and then the graphene oxide was reduced by an electrochemical reduction method. FTIR was used to verify the success of the reduction and the results are shown in Figure 2A. The peaks of GO-Nafion at 3300 cm^−1^, 1729 cm^−1^, 1624 cm^−1^, and 1056 cm^−1^ represented the OH stretching vibration, C = O stretching vibration, C = C stretching vibration, and C-O symmetric vibration, respectively [34]. For the ERGO-Nafion after reduction, the peaks at 3300 cm^−1^ and 1729 cm^−1^ disappeared, and the C = C stretching vibration and C-O at 1624 cm^−1^ and 1056 cm^−1^ became weak, but did not completely disappear. However, it showed that the graphene oxide was reduced, yet a small amount of functional groups (C-O) remained, which was advantageous for improving the biocompatibility of graphene. The SEM image of GO-Nafion (Figure 2B) looked relatively solidified and flat, and there were only some wrinkles, indicating the good film-forming of GO-Nafion. After reduction, the ERGO-Nafion (Figure 2C) showed a more crumpled and wrinkled morphology. AuNPs were electrodeposited by CV for 15 cycles on the surface of GCE/ERGO-Nafion electrodes. It can be clearly seen from Figure 2D that there was a layer of granular particles on the electrode surface, suggesting that the AuNPs were successfully deposited on the electrode surface. Although there were some aggregations of AuNPs, the size of the separate AuNPs was about 58 nm (See Figure S1). These AuNPs provided a large contact area for electron transfer on the electrode surface.

**Figure 3:**
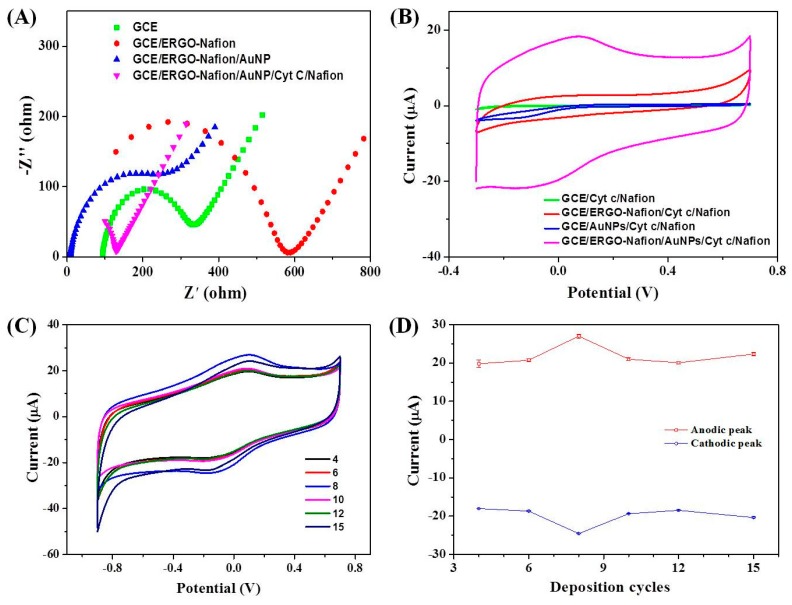
Step-by-step construction of an GCE/ERGO-Nafion/AuNPs/Cyt *c*/Nafion electrode (**A**) Nyquist plot of the EIS spectra of different electrodes in a 10 mM KCl aqueous solution containing 10 mM Fe(CN)_6_^3−^/^4−^(1:1). (**B**) CV curves of different electrodes in a 0.2 M PBS solution (pH = 7) at a scan rate of 50 mV/s. (**C**) CV curves of the GCE/ERGO-Nafion/AuNPs/Cyt *c*/Nafion electrode in a 0.2 M PBS solution (pH = 7) at a scan rate of 50 mV/s with AuNPs deposited by varied cycles. (**D**) Plots of the peak current of the GCE/ERGO-Nafion/AuNPs/Cyt *c*/Nafion electrode against deposition cycles. The relative standard deviation (RSD) of each point is less than 1.0% except the first point of the anodic peak (4.1%).

Cyt *c* and Nafion were deposited by simply being dropped on the surface of the GCE/ERGO-Nafion/AuNPs electrode. EIS technique was employed to support the efficient formation of the GCE/ERGO-Nafion/AuNPs/Cyt *c*/Nafion electrode. Nyquist plots of the EIS spectra of different electrodes in a 100 mM KCl aqueous solution containing 10 mM Fe(CN)_6_^3−^/^4−^(1:1) are shown in Figure 3A. The bare GCE electrode had a small AC impedance (charge transfer resistance, *R*_ct_ = 240 Ω), indicating that the electron transfer speed between the electrolyte and the electron was fast. The modified GCE/ERGO-Nafion electrode had a significantly higher AC impedance (*R*_ct_ = 564 Ω) than the bare GCE electrode, suggesting the electron transfer of the electrode interface of the GCE/ERGO-Nafion electrode was hindered. After AuNPs’ deposition, the GCE/ERGO-Nafion/AuNPs electrode had a smaller AC impedance (*R*_ct_ = 242 Ω) than the GCE/ERGO-Nafion electrode, indicating that the AuNPs promoted electron transfer at the electrode interface and ERGO. Finally, the modified electrode, GCE/ERGO-Nafion/AuNPs/Cyt *c*/Nafion, had a significantly lower AC impedance value (*R*_ct_ = 125 Ω) than the modified electrode GCE/ERGO-Nafion/AuNPs, and even lower than that of the bare GCE electrode, indicating that cytochrome c participated in and promoted the electron transfer at the electrode interface. In addition, it also meant the complex of graphene and gold nanoparticles could keep cytochrome *c* biologically active.

### 3.2. Direct Electrochemistry of GCE/ERGO-Nafion/AuNPs/Cyt c/Nafion Electrode

The direct electrochemistry of cytochrome c on the GCE/ERGO-Nafion/AuNPs/Cyt *c*/Nafion electrode and compared electrodes were explored with CV technique to testify the importance of each of the active materials by comparing the current response of different modified electrodes. As shown in Figure 3B, the GCE/Cyt *c*/Nafion electrode had no redox peak, indicating that the cytochrome *c* did not remain biological activity. The GCE/ERGO-Nafion/Cyt *c*/Nafion electrode had no evident redox peaks, but the capacitance of the electrode increased obviously, due to the addition of ERGO. The GCE/AuNPs/Cyt *c*/Nafion electrode had a weak reduction peak, indicating cytochrome *c* maintained some activity, but this was not very good. The GCE/ERGO-Nafion/AuNPs/Cyt *c*/Nafion electrode had a pair of more obvious redox peaks, the oxidation peak potential was 0.05 V, the reduction peak potential was −0.15 V, and the peak potential difference was 0.2 V. The response current was much larger than that of the other compared electrodes. These results indicated that the complex of ERGO, gold nanoparticles, and Nafion could maintain the biological activity of cytochrome c, and graphene provided a carrier for the loading of gold nanoparticles and cytochrome *c*, and promoted their synergistic effect. 

To study the effect of the deposition amount of gold nanoparticles on the direct electrochemistry of Cyt *c* in the GCE/REGO-Nafion/AuNPs/Cyt *c*/Nafion electrode, the electrodes with varied AuNPs loadings were obtained by controlling the scanning cycles of cyclic voltammetry. CV curves of the GCE/ERGO-Nafion/AuNPs/Cyt *c*/Nafion electrode in a 0.2 M PBS solution (pH = 7) at a scan rate of 50 mV/s with AuNPs deposited by varied cycles are shown in Figure 3C. There were both redox peaks around 0.05 V and −0.15 V in all CV curves, but their peak currents were different. The plots of the peak current of different GCE/ERGO-Nafion/AuNPs/Cyt *c*/Nafion electrodes are shown in Figure 3D. The gold nanoparticles were deposited by cyclic voltammetry. At one, two, and three cycles, the gold nanoparticles were just beginning to deposit, and the amount of gold nanoparticles was relatively small, so it was not shown. The peak current increased first and then decreased with the increase of scan cycles (from four to 15). As the number of scanning cycles increased, more and more gold nanoparticles appeared, and the distance between the nanoparticles became closer and closer, the coverage became higher, and the current became bigger. However, when the scanning cycles increased more than eight, too many gold nanoparticles induced more aggregation, which reduced the surface area for anchoring Cyt *c*, so the current inversely turned smaller. When the number of scanning cycles was eight during electrodeposition, the peak current of the electrode was the largest, indicating that these gold nanoparticles had the best promotion effect on surface electron transfer of the modified GCE/REGO-Nafion/AuNPs/Cyt *c*/Nafion electrode.

**Figure 4:**
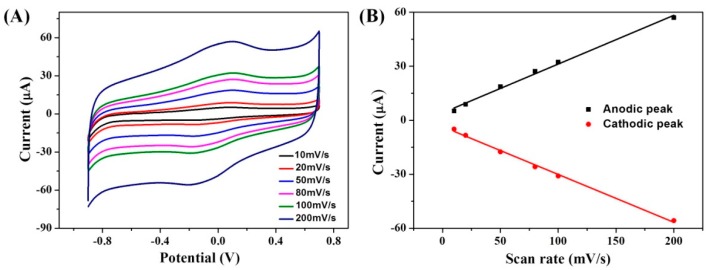
(**A**) CV curves of GCE/ERGO-Nafion/AuNPs/Cyt *c*/Nafion electrodes in 0.2 M PBS (pH = 7.0) at varied scan rates from 10 mV/s to 200 mV/s. (**B**) Plots of redox peak current of GCE/ERGO-Nafion/AuNPs/Cyt *c*/Nafion electrode vs scan rates. The correlation coefficient (R^2^) is 0.9975 and 0.99.85 for the oxidation and reduction peak current, respectively.

The interface reaction control process of the modified electrode determined the rate in the catalytic process, and was also related to the electron transfer of the interface between the modified electrode and the electrochemical performance of the modified electrode. In this study, the control process of the electrode interface was studied at a different scanning rate with a modified GCE/ERGO-Nafion/AuNPs/Cyt *c*/Nafion electrode in a 0.2 M PBS (pH = 7.0) buffer solution. Figure 4A shows the CV curves of the GCE/ERGO-Nafion/AuNPs/Cyt *c*/Nafion electrode at a scan rate of 10, 20, 50, 80, 100, and 200 mV/s in 0.2 M PBS (pH = 7.0), respectively. The peak current increased with the scan rate increasing. Figure 4B displays the dependence between the response current and scan rate. It was found that there was a linear relationship between the peak current of the redox peak and the scan rate at different scan rates from 10 to 200 mV/s. The linear correlation coefficient (R^2^) between the current value of the oxidation peak and the scan rate was 0.9975, and the current value and scan of the reduction peak. The linear correlation coefficient (R^2^) of the rate was 0.9985. This result revealed that the electron transfer of ERGO-Nafion/AuNPs/Cyt *c*/Nafion displayed a surface control rather than a diffusion control process due to the immobilization of Cyt *c*.

### 3.3. Performance of Electrochemical Biosensor based on GCE/ERGO-Nafion/AuNPs/Cyt c/Nafion Electrode

**Figure 5:**
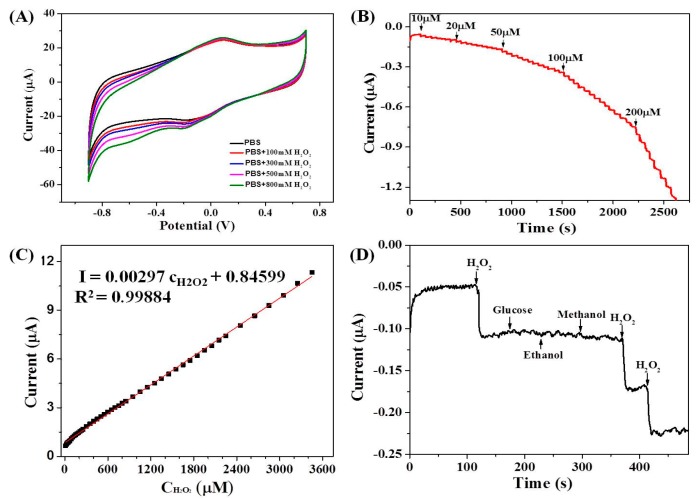
(**A**) CV curves of GCE/ERGO-Nafion/AuNPs/Cyt *c*/Nafion electrode in a 0.2 M PBS (pH = 7.0) containing varied amounts of H_2_O_2_ at a rate of 50 mV/s. (**B**) Typical steady state response of the GCE/ERGO-Nafion/AuNPs/Cyt *c*/Nafion electrode at an applied potential of −0.2 V to the successive injection of H_2_O_2_ into 0.2 M PBS (pH = 7.4). (**C**) Calibration curve of the linear dependence of the cathodic peak current on the H_2_O_2_ concentration. (**D**) Amperometric response of the GCE/ERGO-Nafion/AuNPs/Cyt *c*/Nafion electrode in a 0.2 M PBS (pH = 7.4) at an applied potential of −0.2 V to which 100 μM H_2_O_2_, 100 μM Glucose, 100 μM Ethanol, 100 μM Methanol, 100 μM H_2_O_2_, and 100 μM H_2_O_2_ were added, respectively.

The GCE/ERGO-Nafion/AuNPs/Cyt *c*/Nafion electrode was verified as a biosensor against the H_2_O_2_, and the CVs (Figure 5A) and amperometric responses (Figure 5B) of the GCE/ERGO-Nafion/AuNPs/Cyt *c*/Nafion electrode to H_2_O_2_ were performed. In the CV curves, the reduction peak current at -0.2 V of the biosensor electrode sequentially increased with the increase of H_2_O_2_ concentration. The peak current of the oxidation peak of the modified electrode, GCE/ERGO-Nafion/AuNPs/Cyt *c*/Nafion, had almost no difference in or without the presence of H_2_O_2_ in PBS. The catalytic reaction of the modified GCE/ERGO-Nafion/AuNPs/Cyt *c*/Nafion electrode to H_2_O_2_ was mainly the reduction process. The reaction mechanism was as follows [12,35,36]:

(1) $$\mathrm{Cyt}\;c{\text{-Heme}(\mathrm{Fe}}^{2+})+\frac{1}{2}H_{2}O_{2}{+H}^{+}\to \mathrm{Cyt}\;c{\text{-Heme}(\mathrm{Fe}}^{3+}{)+H}_{2}O$$

(2) $$\mathrm{Cyt}\;c{\text{-Heme}(\mathrm{Fe}}^{3+}{)+e}^{-}\overset{}{\to }\mathrm{Cyt}\;c{\text{-Heme}(\mathrm{Fe}}^{2+})$$

(3) $$\frac{1}{2}H_{2}O_{2}+H^{+}{+e}^{-}\;\overset{\mathrm{Cyt}\;c{\text{-Heme}(\mathrm{Fe}}^{2+/3+})}{\to }H_{2}O$$

Cyt *c*-Heme (Fe^2+^) reduced H_2_O_2_ to H_2_O, and itself converted to Cyt *c*-Heme (Fe^3+^) due to the loss of electrons as in equation (1). Then, Cyt *c*-Heme (Fe^3+^) was rapidly reduced to Cyt c-Heme (Fe^2+^) on the electrode surface. Equation (3) is the general equation for Cyt *c* electrocatalytic H_2_O_2_. As the concentration of H_2_O_2_ increased, the concentration of Cyt *c*-Heme (Fe^3+^) was increased on the surface of the electrode, which led to an increase in the amount of reduction of Cyt *c*-Heme (Fe^2+^) on the surface of the electrode, which increased the change of the reduction peak. Additionally, the oxidation peak changes were not obvious. The change of the reduction peak current verified the direct electron transfer between the active center of heme in the cytochrome *c* and the electrode interface, and the electrocatalytic activity of Cyt *c* on hydrogen peroxide. The modified GCE/ERGO-Nafion/AuNPs/Cyt *c*/Nafion electrode responded to H_2_O_2_ as a reduction process at the electrode interface, and as the concentration of H_2_O_2_ increased, there was a corresponding increase in the response current value at the reduction peak.

To further study the sensitivity, the detection range and the lower detection limit of the modified electrode, GCE/ERGO-Nafion/AuNPs/Cyt *c*/Nafion, upon H_2_O_2_, the amperometric response curve was measured by continuously increasing the concentration of H_2_O_2_. As shown in Figure 5B, upon addition of H_2_O_2_, the reductive current increased, steeply reaching the stable value. The GCE/ERGO-Nafion/AuNPs/Cyt *c*/Nafion electrode displayed a quick electrochemical response toward H_2_O_2_ and achieved 95% of the steady-state current within 4 s. In addition, the electrochemical responses showed a good linear relationship with the H_2_O_2_ concentration ranging from 1.0 × 10^−5^ M to 3.5 × 10^−3^ M (R = 0.999, Figure 5C) with a detection limit of 1.1 × 10^−6^ M at a signal to noise ratio of 3, and the sensitivity was 0.0645 μA/(μM·cm^2^) calculated based on the true surface area of the target electrode (0.04604 cm^2^, the details are shown in Figure S2). The relationship between the current and concentration was I = 0.00297c_H__2__O__2_+0.84599. Results indicated that the modified electrode had satisfactory performance and might be practically applied in the determination of H_2_O_2_ concentrations in blood or food.

The effect of interferences on the H_2_O_2_ response was further studied to evaluate the selectivity of the sensor. Glucose (in blood samples), ethanol, and methanol (in food samples) were common interfering species, and were used as the interference molecules. Figure 5D shows the result of the selectivity of the sensor measured by sequential additions of 100 μM H_2_O_2_, 100 μM Glucose, 100 μM ethanol, 100 μM methanol, 100 μM H_2_O_2_, and 100 μM H_2_O_2_ into the stirring 0.2 M PBS (pH = 7.4). The results indicated that glucose, ethanol, and methanol had no obvious interference in the detection of H_2_O_2_ at the studied concentrations.

**Figure 6:**
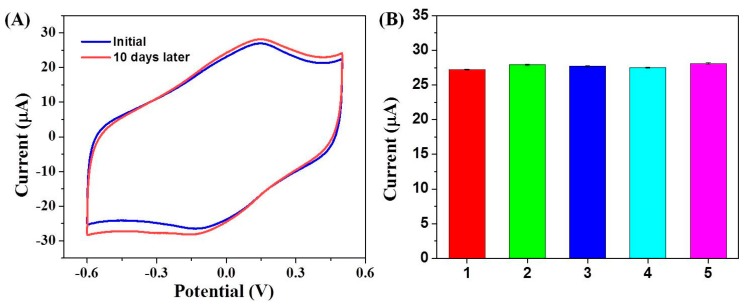
(**A**) CV curves of the GCE/ERGO-Nafion/AuNPs/Cyt c/Nafion electrode as-prepared and stored in PBS at 4 °C 10 days later. (**B**) Amperometric response of five GCE/ERGO-Nafion/AuNPs/Cyt c/Nafion electrodes to 200μM H_2_O_2_ in a 0.2 M PBS (pH = 7.4) at a rate of 50 mV/s. The relative standard deviation (RSD) of each point is less than 0.35%.

After the electrode was stored in N_2_-saturarted PBS (pH = 7.0) at 4 °C for 10 days, the anodic peak current increased by 4.5% and the cathodic peak current increased by 6.4% against the original value (Figure 6A), which indicated the sensor has good stability. The reproducibility was determined from the current response of five independent sensors to 200 μM H_2_O_2_. As shown in Figure 6B, the calculated relative standard deviation value (RSD) was only 1.23%. The good stability and reproducibility were attributed to the good biocompatibility of ERGO-Nafion/AuNPs composite and strong interactions between ERGO-Nafion and AuNPs. Finally, the reliability of the biosensor electrode was performed by measuring the current response upon the addition of different amounts of H_2_O_2_ into a 1.0 mM of H_2_O_2_ solution. The results are summarized in Table 1. The relative deviation of the measured value to the truth value was less than 3%, inferring the great reliability of the biosensor.

The fabrication procedure of our biosensor was simple and the detection limit was comparable to (or lower than) those of most graphene or gold nanoparticles modified electrodes, as shown in Table S1, such as 3.0 μM for Cyt *c* on AuNPs/RTIL/multi-walled carbon nanotubes modified GCE [37], 2.5 μM for Cyt *c* on RTIL-AuNPs-Gp-PDDA/MUA-MCH/Au [25], 3 μM for Cyt *c* on GCE/GNPs/PANS [38], and 1.56 μM for Cyt c on indium tin oxides (ITO)/nanopyramidal gold electrode [29]. Dinesh et al. [18] reported a Nafion/Cyt *c*/GO-CNT/AuNPs/GCE biosensor with a very low detection limit of 0.027 nM, but the linear range was very limited, only about one order of magnitude (0.01–0.14 nM), which indicated that it was restricted in practical applications. The linear range of our biosensor was wide (0.01–3.5 mM). Considering the good selectively, stability, reproducibility, reliability, and relatively low detection limit, the constructed biosensor could have promising applications as the next generation biosensor for H_2_O_2_.

## 4. Conclusions

A cytochrome c-based biosensor denoted as GCE/ERGO-Nafion/AuNPs/Cyt *c*/Nafion was facilely constructed by the dropping-dry method and electrodeposition. The complex of ERGO, gold nanoparticles, and Nafion maintained cytochrome *c* biological activity, and ERGO provided a carrier for the loading of gold nanoparticles and cytochrome c and promoted their synergistic effect. The AuNPs, with an appropriate size, enhanced the synergistic electrocalysis effect on the direct electrochemistry of cytochrome c in the proposed electrode. The constructed biosensor can be utilized for electrocatalytic reduction as well as amperometric determination of H_2_O_2_. The amperometric response showed a linear relationship toward H_2_O_2_ in the concentration range from 0.01 mM to 3.5 mM with a detection limit of 1.1 μM. The biosensor also exhibited high sensitivity and stability with good reproducibility and reliability, which enables them to be used in practical applications in the next generation of biosensors.

## Figures and Tables

**Table 1:** Amperometric response against the addition of different amounts of H_2_O_2_ into a 1 mM H_2_O_2_ solution.

Samples	Initial Concentration (mM)	Addition (mM)	Measured (mM)	Yield (%)
1	1.0	0.1	1.096	99.6
2	1.0	0.4	1.399	99.9
3	1.0	1.0	2.010	100.5
4	1.0	1.5	2.573	102.9

## Supplementary Materials

The following are available online at http://www.mdpi.com/1424-8220/19/1/40/s1, **Figure S1**: Size distribution of the separate gold nanoparticles; **Figure S2**: The CV curves of GCE/ERGO-Nafion/AuNPs/Cyt *c*/Nafion electrode in 100 mM KCl solution containing 10 mM of K_3_[Fe(CN)_6_]/K_4_[Fe(CN)_6_] (1:1) (A), Plots of oxidation peak current of modified electrode vs scan rates (B); **Table S1**: Comparison analytical performance of some Cyt *c*-based H_2_O_2_ biosensors.

## References

[1] Wang J., Li M., Shi Z., Li N., Gu Z. (2002). Direct electrochemistry of cytochrome c at a glassy carbon electrode modified with single-wall carbon nanotubes. Anal. Chem..

[2] Tahirov T.H., Misaki S., Meyer T.E., Cusanovich M.A., Higuchi Y., Yasuoka N. (1996). High-resolution Crystal Structures of Two Polymorphs of Cytochromec′ from the Purple Phototrophic BacteriumRhodobacter capsulatus. J. Mol. Biol..

[3] Ren Z., Meyer T., McRee D.E. (1993). Atomic Stucture of a Cytochrome c′ with an Unusual Ligand-controlled Dimer Dissociation at 1· 8 Å Resolution. J. Mol. Biol..

[4] Caffrey M., Simorre J.-P., Brutscher B., Cusanovich M., Marion D. (1995). NMR assignment of Rhodobacter capsulatus ferricytochrome c′, a 28 kDa paramagnetic heme protein. Biochemistry.

[5] Alvarez-Paggi D.N., Hannibal L., Castro M.A., Oviedo-Rouco S., Demicheli V., Tortora V., Tomasina F., Radi R., Murgida D.H. (2017). Multifunctional cytochrome c: Learning new tricks from an old dog. Chem. Rev..

[6] Davis K.L., Drews B.J., Yue H., Waldeck D.H., Knorr K., Clark R.A. (2008). Electron-transfer kinetics of covalently attached cytochrome c/SAM/Au electrode assemblies. J. Phys. Chem. C.

[7] Aghamiri Z.S., Mohsennia M., Rafiee-Pour H.-A. (2018). Immobilization of cytochrome c and its application as electrochemical biosensors. Talanta.

[8] Balamurugan A., Chen S.-M. (2008). Fabrication of cytochrome c-poly (5-amino-2-napthalenesulfonic acid) electrode by one step procedure and direct electrochemistry of cytochrome c. Biosens. Bioelectron..

[9] Wang G., Xu J.-J., Chen H.-Y. (2002). Interfacing cytochrome c to electrodes with a DNA–carbon nanotube composite film. Electrochem. Commun..

[10] Armstrong F.A., Hill H.A.O., Oliver B.N., Walton N.J. (1984). Direct electrochemistry of redox proteins at pyrolytic graphite electrodes. J. Am. Chem. Soc..

[11] Haymond S., Babcock G.T., Swain G.M. (2002). Direct electrochemistry of cytochrome c at nanocrystalline boron-doped diamond. J. Am. Chem. Soc..

[12] Aghamiri Z.S., Mohsennia M., Rafiee-Pour H.-A. (2018). Fabrication and characterization of cytochrome c-immobilized polyaniline/multi-walled carbon nanotube composite thin film layers for biosensor applications. Thin Solid Films.

[13] Xu X., Yan S., Wang B., Qu P., Wang J., Wu J. (2016). Graphene aerogel/platinum nanoparticle nanocomposites for direct electrochemistry of cytochrome c and hydrogen peroxide sensing. J. Nanosci. Nanotechnol..

[14] Kafi A., Yusoff M., Choucair M., Crossley M.J. (2017). A conductive crosslinked graphene/cytochrome c networks for the electrochemical and biosensing study. J. Solid State Electrochem..

[15] Liao L., Peng H., Liu Z. (2014). Chemistry makes graphene beyond graphene. J. Am. Chem. Soc..

[16] Shao Y., Wang J., Wu H., Liu J., Aksay I.A., Lin Y. (2010). Graphene based electrochemical sensors and biosensors: A review. Electroanalysis.

[17] Lawal A.T. (2015). Synthesis and utilisation of graphene for fabrication of electrochemical sensors. Talanta.

[18] Dinesh B., Mani V., Saraswathi R., Chen S.-M. (2014). Direct electrochemistry of cytochrome c immobilized on a graphene oxide–carbon nanotube composite for picomolar detection of hydrogen peroxide. RSC Adv..

[19] Yan L., Zheng Y.B., Zhao F., Li S., Gao X., Xu B., Weiss P.S., Zhao Y. (2012). Chemistry and physics of a single atomic layer: Strategies and challenges for functionalization of graphene and graphene-based materials. Chem. Soc. Rev..

[20] Tao C.A., Zou X., Hu Z., Liu H., Wang J. (2016). Chemically functionalized graphene/polymer nanocomposites as light heating platform. Polym. Compos..

[21] Tao C.A., Wang J., Qin S., Lv Y., Long Y., Zhu H., Jiang Z. (2012). Fabrication of pH-sensitive graphene oxide–drug supramolecular hydrogels as controlled release systems. J. Mater. Chem..

[22] Alwarappan S., Joshi R.K., Ram M.K., Kumar A. (2010). Electron transfer mechanism of cytochrome c at graphene electrode. Appl. Phys. Lett..

[23] Wang G.-X., Qian Y., Cao X.-X., Xia X.-H. (2012). Direct electrochemistry of cytochrome c on a graphene/poly (3, 4-ethylenedioxythiophene) nanocomposite modified electrode. Electrochem. Commun..

[24] Zhang N., Lv X., Ma W., Hu Y., Li F., Han D., Niu L. (2013). Direct electron transfer of cytochrome c at mono-dispersed and negatively charged perylene–graphene matrix. Talanta.

[25] Song Y., Liu H., Wan L., Wang Y., Hou H., Wang L. (2013). Direct Electrochemistry of Cytochrome c Based on Poly (diallyldimethylammonium Chloride)-Graphene Nanosheets/Gold Nanoparticles Hybrid Nanocomposites and Its Biosensing. Electroanalysis.

[26] Bas S.Z. (2015). Gold nanoparticle functionalized graphene oxide modified platinum electrode for hydrogen peroxide and glucose sensing. Mater. Lett..

[27] Wang L., Deng M., Ding G., Chen S., Xu F. (2013). Manganese dioxide based ternary nanocomposite for catalytic reduction and nonenzymatic sensing of hydrogen peroxide. Electrochim. Acta.

[28] Xie L., Xu Y., Cao X. (2013). Hydrogen peroxide biosensor based on hemoglobin immobilized at graphene, flower-like zinc oxide, and gold nanoparticles nanocomposite modified glassy carbon electrode. Colloids Surf. B.

[29] Liu H., Tian Y., Deng Z. (2007). Morphology-dependent electrochemistry and electrocatalytical activity of cytochrome c. Langmuir.

[30] Lv Y., Tao C.-A., Wang J., Long Y., Zhu H., Tong X., Li H., Zhang Y. (2013). Study of Influence of Acid Ratios in the Oxidation Process on the Structures of Chemically Converted Graphene. Graphene.

[31] Lv Y., Wang F., Zhu H., Zou X., Tao C.-A., Wang J. (2016). Electrochemically reduced graphene oxide-nafion/Au nanoparticle modified electrode for hydrogen peroxide sensing. Nanomater. Nanotechnol..

[32] Yin C., Tao C.A., Cai F., Song C., Gong H., Wang J. (2016). Effects of activation temperature on the deoxygenation, specific surface area and supercapacitor performance of graphene. Carbon.

[33] Tao C.A., Zhang H., Huang J., Zou X., Zhu H., Wang J. (2017). Reduction versus cross-linking: How to improve the tensile strength of graphene oxide/polyvinyl alcohol composite film. Mater. Res. Express.

[34] Liu H.P., Zhan G.Y., Dong Q.Z., Lv Y.A., Wang J.F., Tao C.A., Hu Z.H. (2013). Glucose Biosensor Based on Pt Nanoparticles/Graphene Chitosan Bionanocomposites. Appl. Mech. Mater..

[35] Yagati A.K., Lee T., Min J., Choi J.-W. (2012). Electrochemical performance of gold nanoparticle–cytochrome c hybrid interface for H_2_O_2_ detection. Colloid Surf. B.

[36] Sheng Q.-L., Zheng J.-B., Shang-Guan X.-D., Lin W.-H., Li Y.-Y., Liu R.-X. (2010). Direct electrochemistry and electrocatalysis of heme-proteins immobilized in porous carbon nanofiber/room-temperature ionic liquid composite film. Electrochim. Acta.

[37] Xiang C., Zou Y., Sun L.-X., Xu F. (2008). Direct electron transfer of cytochrome c and its biosensor based on gold nanoparticles/room temperature ionic liquid/carbon nanotubes composite film. Electrochem. Commun..

[38] Feng W., Ji P. (2011). Enzymes immobilized on carbon nanotubes. Biotechnol. Adv..

