# Supplementary material for: Construction of a Biosensor Based on a Combination of Cytochrome c, Graphene, and Gold Nanoparticles
Source: Sensors (Basel). 2018 Dec 22;19(1):40. doi: 10.3390/s19010040 (PMC6339241; doi:10.3390/s19010040)
Supplement: Supplementary file 1 [file sensors-19-00040-s001.pdf]

Construction of a biosensor based on a combination of cytochrome c, electrochemical reduced graphene oxides and gold nanoparticles

Chenxing Guo[1,2†], Jianfang Wang [2†*], Xianzhe Chen[2], Yujiao Li[2], Lifang Wu[1], Jin Zhang[1*] and Cheng-an Tao[2*]

[1] Key Laboratory of Environmentally Friendly Chemistry and Application of Ministry of Education, College of Chemistry, Xiangtan University, Xiangtan 411105, China; 1143757046@qq.com; 1522803862@qq.com

[2] College of Liberal Arts and Science, National University of Defense Technology，Changsha 410073, China; chenxianzhe13@nudt.edu.cn; liyujiao@nudt.edu.cn

[†] These authors contribute to this work equally.

* Correspondence: wangjianfang@nudt.edu.cn (J.W.), Tel.: +86-731-87001801 (J.W.); tccdzdc@163.com (J.Z.); tca02@mails.thu.edu.cn (C.T.)

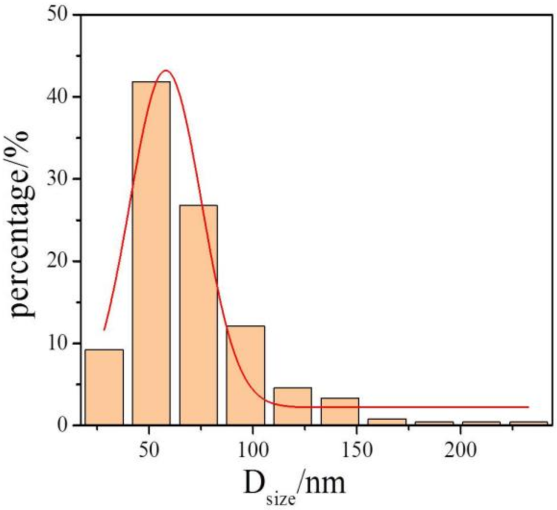

Figure S1. Size distribution of the separate gold nanoparticles

The true surface area of the electrode was calculated through a current of CV plot at different scan rates (10-100 mV/s) in 100 mM KCl solution containing 10 mM of $K_3[Fe(CN)_6]/K_4[Fe(CN)_6]$ (1:1). The CV curves of modified electrode versus scan rates were shown in Figure S2A. Plot of oxidation peak current of modified electrode against root of scan rates is shown in Figure S2B. The correlation coefficient (R^2) was 0.99.07. The surface area of the target sensor is calculated according to the Randles-Savcik formula, $i_p = 2.69 \times 10^5 A n^{5/2} {D_0}^{1/2} v^{1/2} C_0$, where n is the number of electron transfer involved in the redox reaction (n=1 for $[Fe(CN)_6]^{3-/4-}$ solution); A represents the true surface area of the electrode surface; D_0 represents the diffusion coefficient (for $[Fe(CN)_6]^{3-/4-}$ solution: $D_0 = 0.673 \times 10^{-5}$ cm^2/s); ν represents the scanning rate of cyclic voltammetry; C_0 represents the volume concentration of the probe; i_p represents the current value of the redox peak. After calculation, the true surface area of the target electrode is 0.04604 cm^2.

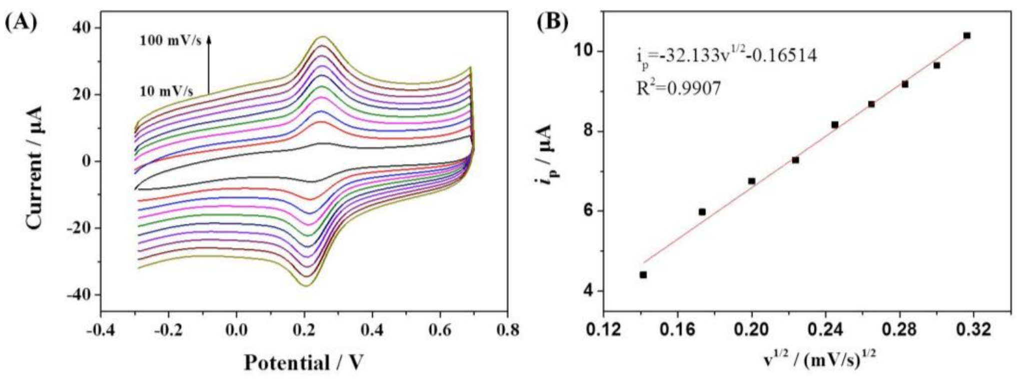

Figure S2 The CV curves of GCE/ERGO-Nafion/AuNPs/Cyt c/Nafion electrode in 100 mM KCl solution containing 10 mM of $K_3[Fe(CN)_6]/K_4[Fe(CN)_6]$ (1:1) (A), Plots of oxidation peak current of modified electrode vs scan rates (B).

Table S1. Comparison of the analytical performance of some Cyt c-based H_2O_2 biosensors.

No.	Various modified electrodes	Detection limit (μM)	Linear range (mM)	Reference
1	Cyt c/nanoporous Au film	6.3	0.01-12	[1]
2	Cyt c/RTIL-PDDA-AuNPs/MUA-MCH/Au	5.0	0.04–3.45	[2]
3	Cyt c/RTIL-PDDA-graphene/MUA-MCH/Au	2.5	0.02–3.45	
4	Cyt c/RTIL-AuNPs-graphene -PDDA/MUA-MCH/Au	2.5	0.01–4.45	
5	Cyt c/Nanorod-like gold/ITO	3.70	0.050-1.5	[3]
6	Cyt c/Nanopyramidal gold/ITO	1.56	0.010-1	[3]
7	Cyt c/PTCA-graphene/GCE	3.5	0.005-0.09	[4]
8	Cyt c/AuNPs/RTIL/MWNTs/GCE	3.0	0.05–1.15	[5]

No.	Various modified electrodes	Detection limit (μM)	Linear range (mM)	Reference
**9**	**Nafion/Cyt c/AuNPs/ERGO-Nafion/GCE**	**1.1**	**0.01-3.5**	**This work**
10	Cyt c/MWCNTs/CF/GCE	1.0	0.002-0.078	[6]
11	Cyt c/AuNPs/Chit/MWNTs/GCE	0.91	0.0015-0.51	[7]
12	Cyt c/11-MUA/AuNPs/3-MPTMS/ITO	0.5	---	[8]
13	Cyt c/NaY/GCE	0.32	0.008-0.128	[9]
14	Cyt c/MWCNT-PANI/ITO	0.3	0.0005-1.5	[10]
15	Cyt c/Graphene-PEDOT/GCE	0.249	0.0005-0.4	[11]
16	Cyt c/L-Cys/P3MT/MWCNT/GCE	0.23	0.0007-0.4	[12]
17	Nafion/Cyt *c*/GO-CNT/AuNPs/GCE	0.000027	$1x10^{-8}$ -$1.4x10^{-7}$	[13]

Cytochrome c (Cyt c)

room temperature ionic liquid (RTIL)

poly(diallyldimethylammonium chloride) (PDDA)

gold nanoparticles (AuNPs)

11-mercaptoundecanoic acid-6-mercapto-1-hexanol (MUA-MCH)

gold electrode (Au)

indium tin oxide (ITO)

3,4,9,10-perylenetetracarboxylicacid(PTCA)

glassy carbon electrode(GCE)

multi-walled carbon nanotubes (MWNTs)

electrochemical reduced graphene oxides (ERGO)

ciprofloxacin (CF)

chitosan (Chit)

11-mercapundecanoic acid (11-MUA)

3-mercaptopropyl trimethoxysilane (3-MPTMS).

Zeolite (NaY)

Polyaniline (PANI)

poly(3,4-ethylenedioxythiophene) (PEDOT)

L-Cystine (L-Cys)

poly(3-methylthiophene) (P3MT)

graphene oxides (GO)

carbon nanotubes (CNT)

[1] Zhu, A.; Tian, Y.; Liu, H.; Luo, Y., Nanoporous gold film encapsulating cytochrome c for the fabrication of a H_2O_2 biosensor. *Biomaterials* **2009**, *30*, 3183-3188.

[2] Song, Y.; Liu, H.; Wan, L.; Wang, Y.; Hou, H.; Wang, L., Direct electrochemistry of cytochrome c based on poly (diallyldimethylammonium chloride) ‑ graphene nanosheets/gold nanoparticles hybrid nanocomposites and its biosensing. *Electroanalysis* **2013**, *25*, 1400-1409.

[3] Liu, H.; Tian, Y.; Deng, Z., Morphology-dependent electrochemistry and electrocatalytical activity of cytochrome c. *Langmuir* **2007**, *23*, 9487-9494.

[4] Zhang, N.; Lv, X.; Ma, W.; Hu, Y.; Li, F.; Han, D.; Niu, L., Direct electron transfer of cytochrome c at mono-dispersed and negatively charged perylene–graphene matrix. *Talanta* **2013**, *107*, 195-202.

[5] Xiang, C.; Zou, Y.; Sun, L.-X.; Xu, F., Direct electron transfer of cytochrome c and its biosensor based on gold nanoparticles/room temperature ionic liquid/carbon nanotubes composite film. *Electrochemistry Communications* **2008**, *10*, 38-41.

[6] Kumar, S. A.; Wang, S.-F.; Yeh, C.-T.; Lu, H.-C.; Yang, J.-C.; Chang, Y.-T., Direct electron transfer of cytochrome c and its electrocatalytic properties on multiwalled carbon nanotubes/ciprofloxacin films. *Journal of Solid State Electrochemistry* **2010**, *14*, 2129-2135.

[7] Xiang, C.; Zou, Y.; Sun, L.; Xu, F., Direct electrochemistry and electrocatalysis of cytochrome c immobilized on gold nanoparticles-chitosan-carbon nanotubes-modified electrode. *Talanta* **2007**, *74*, 206-211.

[8] Yagati, A. K.; Lee, T.; Min, J.; Choi, J.-W., Electrochemical performance of gold nanoparticle–cytochrome c hybrid interface for h2o2 detection. *Colloids and Surfaces B: Biointerfaces* **2012**, *92*, 161-167.

[9] Dai, Z.; Liu, S.; Ju, H., Direct electron transfer of cytochrome c immobilized on a nay zeolite matrix and its application in biosensing. *Electrochimica Acta* **2004**, *49*, 2139-2144.

[10] Lee, K.-P.; Gopalan, A. I.; Komathi, S., Direct electrochemistry of cytochrome c and biosensing for hydrogen peroxide on polyaniline grafted multi-walled carbon nanotube electrode. *Sensors and Actuators B: Chemical* **2009**, *141*, 518-525.

[11] Wang, G.-X.; Qian, Y.; Cao, X.-X.; Xia, X.-H., Direct electrochemistry of cytochrome c on a graphene/poly (3, 4-ethylenedioxythiophene) nanocomposite modified electrode. *Electrochemistry Communications* **2012**, *20*, 1-3.

[12] Eguilaz, M.; Agüí L.; Yanez-Sedeno, P.; Pingarron, J., A biosensor based on cytochrome c immobilization on a poly-3-methylthiophene/multi-walled carbon nanotubes hybrid-modified electrode. Application to the electrochemical determination of nitrite. *Journal of Electroanalytical Chemistry* **2010**, *644*, 30-35.

[13] Dinesh, B.; Mani, V.; Saraswathi, R.; Chen, S.-M., Direct electrochemistry of cytochrome c immobilized on a graphene oxide–carbon nanotube composite for picomolar detection of hydrogen peroxide. *RSC Advances* **2014**, *4*, 28229-28237.
